# Exploratory characterization of IgG1/IgG4 glycosylation and monocyte-derived dendritic cell responses in esophageal squamous cell carcinoma

**DOI:** 10.3389/fimmu.2026.1832782

**Published:** 2026-06-18

**Authors:** Hui Wang, Jun Li, Yinghai Wang, Yuebin Shi, Peiren Tang, Ying Li, Jiang Gu, Li Wang

**Affiliations:** 1 Department of Pathology, The First People’s Hospital of Yunnan Province, Kunming University of Science and Technology Affiliated Hospital, Kunming, China; 2 Sample Biobank, National Biobank of China, Shenzhen Medical Academy of Research and Translation (SMART), Shenzhen, China; 3 Department of Gynecology, Peking University Cancer Hospital Yunnan, Yunnan Cancer Hospital, The Third Affiliated Hospital of Kunming Medical University, Kunming, China; 4 Provincial Key Laboratory of Molecular Pathology and Personalized Medicine Center of Collaborative and Creative Center, Department of Pathology and Pathophysiology, Shantou University Medical College, Shantou, China; 5 Jinxin Research Institute for Reproductive Medicine and Genetics, Xinan Hospital for Maternal and Child Health Care, Chengdu, China

**Keywords:** dendritic cell, differentiation, glycosylation, immunoglobulin G1, immunoglobulin G4

## Abstract

**Introduction:**

In previous studies, we identified the unique tumor immunosuppressive function of IgG4. This study aims to further characterize the structure of IgG4 and evaluate its potential effects on monocyte-derived dendritic cells (moDCs).

**Methods:**

The densities of IgG4 and CD11c in esophageal cancer tissues were detected by immunohistochemistry. IgG1 and IgG4 subclasses were purified from IgG1 and IgG4 subclasses were purified from intravenous immunoglobulin (IVIg) and human serum samples using subclass‑specific affinity chromatography. Human peripheral CD14⁺ monocytes were isolated and induced to differentiate into moDCs *in vitro*. The migratory and phagocytic capacities of moDCs were then evaluated following stimulation with IgG1 and IgG4.

**Results:**

Immunohistochemical staining revealed that the densities of IgG4⁺ and CD11c⁺ cells were significantly higher in esophageal squamous cell carcinoma (ESCC) tissues than in adjacent normal tissues. Protein electrophoresis, molecular weight analysis, lectin affinity chromatography, and spectrometry demonstrated that native IgG4 exhibits distinct glycosylation profiles compared with IgG1 or commercial myeloma-derived IgG4. IgG4 was predominantly enriched in the ConA⁺ IgG fraction, indicating that it undergoes extensive high-mannose glycosylation. Immunocytochemical staining revealed that peripheral blood-derived monocytes expressed CD68 and CD206, and displayed strong binding affinity for IgG1 but weak affinity for IgG4. *In vitro* co-culture of moDCs with IgG4 revealed that IgG4 significantly promoted the migration and phagocytic capacity of moDCs relative to IgG1. Furthermore, IgG4 purified from both IVIg and patient serum exerted comparable effects on moDC differentiation.

**Discussion:**

Collectively, these findings suggest that IgG4-associated glycosylation features may be linked to altered phenotypic and functional parameters of moDCs in this exploratory *in vitro* model. However, the underlying mechanisms, tissue-level relevance, and biological significance require further validation in larger cohorts and more comprehensive functional studies.

## Introduction

As the least abundant subclass of immunoglobulin G (IgG), IgG4 mediates unique functions in human humoral immunity. Its distinct structural and molecular characteristics closely link IgG4 to a variety of clinical disorders, particularly autoimmune and allergic diseases (1). IgG4 is a typical Y-shaped immunoglobulin composed of two γ4 heavy chains and two κ or λ light chains linked by disulfide bonds, with an approximate molecular weight of 150 kDa. Each IgG4 molecule contains two antigen-binding Fab arms and one Fc region. Its heavy chain is encoded by the IGHG4 gene on chromosome 14. This gene is tandemly arranged with IGHG1, IGHG2 and IGHG3 genes, and IgG4 is converted from IgM or IgA via class switch recombination (CSR) (2, 3). The currently identified structural characteristics of IgG4 are as follows: Firstly, the heavy chain constant region of IgG4 is composed of three domains, namely CH1, CH2 and CH3. Compared with IgG1 and IgG3, IgG4 generally exhibits reduced binding affinity for Fcγ receptors and complement component C1q, resulting in limited activation of Fc-mediated effector functions and the classical complement pathway. This relatively low effector function is one of the key reasons why IgG4 has been widely used as a backbone for therapeutic antibodies when immune activation is not desired (3). Secondly, IgG4 has a relatively short hinge region with a unique disulfide bond linkage pattern. This structural property endows the molecule with greater conformational flexibility, but also results in relatively low antigen-binding affinity (4). Thirdly, IgG4 exhibits a unique Fab-arm exchange capability (5). The distinctive amino acid composition of the IgG4 hinge region enables the formation of intrachain disulfide bonds, which induces the dissociation of interchain disulfide bonds in the hinge region of the two heavy chains and triggers the phenomenon of Fab arm exchange (FAE). Specifically, the heavy chain-light chain complexes (half-molecules) of two IgG4 molecules undergo exchange, generating bispecific antibodies with two distinct antigen-binding sites. This characteristic prevents the formation of stable antigen-antibody complexes, thereby further attenuating the intensity of immune responses. Furthermore, IgG4 possesses an astonishing Fc-Fc binding ability (6, 7). IgG4 exhibits non-specific affinity for other IgG subclasses and can undergo conformational changes via Fc-Fc interactions.

IgG4 has a diminished capacity to engage FcγR and C1q, which renders it unable to efficiently trigger antibody-dependent cell-mediated cytotoxicity (ADCC) and complement-dependent cytotoxicity (CDC), thus exerting only a weak immune effector function. IgG4 binds to target antigens and competitively blocks the binding of high-affinity IgG subclasses such as IgG1, which in turn attenuates excessive immune reactions and underpins its role as a blocking antibody in the context of allergic diseases (8).

IgG4-related disease (IgG4-RD) is the clinical disorder most intimately associated with IgG4. As a systemic autoimmune condition, it is characterized by significantly elevated serum IgG4 concentrations, tissue infiltration by IgG4-positive plasma cells, and prominent fibrosis, with the potential to involve multiple organ systems. Frequently affected organs include the pancreas, bile ducts, lacrimal and salivary glands, kidneys, and lymph nodes, among others. In addition, elevated serum IgG4 levels have been documented in rheumatoid arthritis and various allergic disorders. In recent years, increased IgG4 expression has been observed in a wide range of tumor tissues including malignant melanoma (9), extrahepatic cholangiocarcinoma (10), esophageal cancer (11) and gastric cancer (12), and has been associated with unfavorable patient prognosis. The unique characteristics of IgG4, including its low antigen-binding affinity, Fab-arm exchange, and Fc-Fc interactions, have been implicated in the suppression of anti-tumor immune responses (13–15).

Esophageal cancer exhibits a notably high prevalence in the Chaoshan area of Guangdong Province, China. In the Chinese population, esophageal squamous cell carcinoma (ESCC) accounts for 85.79% of all esophageal cancer cases. Esophageal cancer mainly comprises two major histological subtypes: ESCC and esophageal adenocarcinoma (EAC). These two subtypes differ substantially in their pathogenesis, epidemiological profiles, biological behavior, therapeutic strategies, and prognosis. Compared with EAC, ESCC is characterized by higher surgical risks, impaired postoperative quality of life, and inferior overall survival rates (16). Over the past decade, the incidence and mortality rates of esophageal cancer have been continuously decreasing due to novel adjuvant therapy and immunotherapy, however, a subset of patients still exhibits a poor response to treatment, and the overall disease burden remains heavy (17). The main risk factors for ESCC include smoking, alcohol consumption, low activity of alcohol-metabolizing enzymes ALDH1/2, and human papillomavirus (HPV) infection (18). Consumption of overheated liquids, nutritional deficiencies, and low intake of fruits and vegetables are also recognized risk factors. Additionally, dysregulations in epigenetic regulatory processes, cell cycle-related genes, and signaling pathways such as NOTCH, WNT, and TP53 have been implicated in the initiation and progression of ESCC (19). Traditionally, the treatment of ESCC has relied primarily on radiotherapy, chemotherapy, surgical resection, or a combination of these modalities. All the aforementioned approaches are invasive and have failed to significantly improve patient survival for years (20). In recent years, studies have found extensive infiltration of tertiary lymphoid structures in esophageal cancer tissues (21), which is associated with tumor prognosis and immunotherapy (22). Detailed analysis of the immune microenvironment in esophageal cancer will facilitate the understanding of the disease and the development of novel therapies.

First discovered by Ralph Steinman in 1973, DCs are a class of bone marrow-derived professional antigen-presenting cells (23, 24). In humans, DCs are mainly classified into two subsets: one is myeloid DCs (mDCs), which express the surface marker CD11c and can be further subdivided into CD1c⁺ DCs and CD141⁺ DCs. The other is plasmacytoid DCs (pDCs), characterized by the surface marker CD123 (25–28). Upon capturing antigens, DCs migrate to the draining lymph nodes, present the processed and modified antigens to T cells, and thereby initiate the adaptive immune response. DCs can also exert their functions either directly through interactions with B cells or indirectly by promoting the expansion and differentiation of CD4⁺ T cells (29, 30). Therefore, DCs act as a bridge linking humoral immunity and cellular immunity (31, 32). Tumor rejection requires the involvement of DCs (33, 34). Accumulating evidence has shown that in esophageal cancer and other malignancies, tumor-infiltrating DCs (especially mDCs) play a certain role in the tumor microenvironment (34–37) and correlate with clinical prognosis (38, 39). However, conflicting findings have been reported in different studies, and the exact pro- or anti-tumor role of tumor-infiltrating DCs remains controversial (40).

Our previous work demonstrated that IgG4 expression is upregulated in ESCC and correlates negatively with patient prognosis (41). Moreover, this upregulation is accompanied by the infiltration of immune cells such as macrophages. The weak antigen-binding affinity of IgG4, along with the weak ADCC and antibody-dependent cellular phagocytosis (ADCP) effects mediated by IgG4, are potential mechanisms that promote tumor growth. By virtue of Fc-Fc interference, IgG4 can compete with IgG1 for binding, thereby impairing multiple immune responses including ADCC, CDC and ADCP (11, 42, 43). In addition, our research group has previously investigated the interaction mechanism between IgG4 and macrophages (42, 44). Given that IgG4 exerts diverse modes of action in its interactions with immune cells, and as a component of humoral immunity, a key question arises: does IgG4 engage in cross-talk with DC-mediated adaptive immunity, especially T-cell immunity? Furthermore, can IgG4 affect the phagocytosis, migration, antigen presentation and other core functions of DCs? Existing studies have shown that IgG binding to DCs can elicit anti-tumor responses (45), with CD11c⁺ mDCs exerting the most prominent effects. However, research on the relationship between IgG4 and DCs remains relatively scarce. Therefore, the potential influence of IgG4 on the differentiation and functional properties of DCs requires further investigation.

## Materials and methods

### Sample collection

Surgical specimens of esophageal squamous cell carcinoma (ESCC) tissues, paired paracancerous tissues, and normal control tissues were obtained from 29 patients at the Affiliated Cancer Hospital of Shantou University Medical College. Peripheral blood samples were collected from 10 healthy adult volunteers and esophageal cancer patients. Peripheral blood cells from healthy donors were used to isolate CD14⁺ monocytes. Serum samples from healthy donors and patients were used to purify total IgG, IgG1, and IgG4, respectively. Because the volume of patient serum was limited, patient serum samples were pooled before purification to minimize inter-sample variability and to obtain sufficient amounts of IgG, IgG1, and IgG4 for subsequent experiments. All ESCC diagnoses were histopathologically confirmed by the Department of Pathology. The human ESCC cell lines KYSE-150 and KYSE-510 were obtained from the laboratory cell bank of Provincial Key Laboratory of Molecular Pathology and Personalized Medicine Center of Collaborative and Creative Center. This study was approved by the Ethics Committee of Shantou University Medical College, and written informed consent was obtained from all participants.

### Hematoxylin and eosin staining

Paraffin-embedded tissue sections were dried in an oven at 65°C for approximately 10 min, followed by deparaffinization through sequential immersion in xylene (I–III, 10 min each). The sections were then rehydrated through a graded ethanol series (absolute ethanol I and II, 10 min each; 95%, 85%, and 75% ethanol, 5 min each), rinsed in double-distilled water for 5 min, and equilibrated in phosphate-buffered saline (PBS) for an additional 5 min. Sections were stained with hematoxylin for 5 min and rinsed thoroughly with running tap water for 5 min. Differentiation was performed using 1% hydrochloric acid–ethanol for 2–3s, followed by washing with water for 10 min. The sections were then counterstained with eosin for approximately 1 min, dehydrated through graded ethanol, cleared in xylene, and mounted using neutral mounting medium with coverslips.

### Immunohistochemistry

Deparaffinization and rehydration were performed as described for H&E staining. Endogenous peroxidase activity was blocked by incubating the sections in 3% hydrogen peroxide at room temperature in the dark for 10–15 min. Antigen retrieval was carried out using sodium citrate buffer (pH 6.0) or Tris–EDTA buffer (pH 9.0) with microwave heating. Sections were heated for 2.5 min, followed by a 2-min interval and an additional 20 s of heating; this cycle was repeated 5–6 times. Sections were then allowed to cool naturally to room temperature. Non-specific binding was blocked by incubation with 10% horse serum for 1 h at room temperature. Primary antibodies were diluted according to the manufacturers’ instructions (**Supplementary Table 1**) and incubated with the sections overnight in a humidified chamber. Sections were washed three times in PBS (5 min each), then incubated with PV9000 Reagent I at 37 °C for 20 min. After PBS rinsing, sections were treated with PV9000 Reagent II at 37 °C for 30 min. Chromogenic development was performed using AEC substrate, and the reaction was terminated once optimal staining intensity was achieved. Sections were counterstained with hematoxylin, mounted with glycerol gelatin, and scanned using a digital slide scanning system.

### Purification of IgG1 and IgG4

Human IgG1/IgG4 affinity agarose (1mL) was added to a Poly−Prep chromatography column. After complete drainage of the column liquid, the column was washed 2–3 times with 5 mL of PBS. An appropriate volume of protein sample was loaded onto the column, with the total protein amount not exceeding the maximum binding capacity (8 mg/mL for IgG1 and 6 mg/mL for IgG4). Following sample loading, the column was washed again 2–3 times with 5 mL of PBS. The target protein was eluted with 5 mL of elution buffer, and this elution step was repeated once; the eluates were combined and collected immediately. Subsequently, 1 M Tris Base was added at a volume equivalent to 10% of the collected eluate to adjust the pH to 7.0. The column was re−equilibrated by washing twice with 5 mL of PBS, followed by the addition of 5 mL of binding buffer for short−term storage. The combined eluate was transferred to a 15 mL centrifugal concentrator with a 10 kDa molecular weight cut−off (MWCO) membrane. The sample was centrifuged at 10, 000 rpm and 4 °C for 10 min, and the filtrate in the lower collection tube was discarded. An appropriate volume of PBS was added to the upper concentrator tube, and the centrifugation step was repeated 2–3 times for buffer exchange. The sample was concentrated to a high concentration for subsequent storage. The concentrated IgG solution was collected from the upper tube, and the protein concentration was determined using a BCA protein assay kit. The purified IgG solution was stored at 4 °C for short−term use, or supplemented with 5% trehalose as a cryoprotectant and stored at −20 °C for long−term preservation. The purity of the isolated IgG1 and IgG4 subclasses was assessed by Western blotting, and the reagents used are listed in **Supplementary Table 2**.

### Isolation of peripheral blood mononuclear cells

Prior to sample collection, written informed consent was obtained from all blood donors. Venous blood was collected from healthy adult donors with no history of major diseases or surgeries, no flu-like symptoms (including cough, expectoration, fever, or rhinorrhea), and no medication use within the previous month. Blood was drawn into lithium heparin anticoagulant tubes, and a small volume of venous blood was also collected into additive-free tubes. Whole blood samples were centrifuged at 400×g for 10 min at room temperature. After removal of the supernatant, the residual blood was diluted with two volumes of PBS. Subsequently, 15 mL of Ficoll solution was gently added to the bottom of a 50 mL rapid lymphocyte separation tube beneath the barrier layer. Subsequently, the diluted blood was gently overlaid on top of the Ficoll layer. Centrifugation was carried out at 1200×g for 10 minutes at 19 °C, with the acceleration and deceleration parameters set to grade 6. The liquid above the white cell layer was carefully aspirated using a Pasteur pipette, and the remaining liquid was transferred to a 50 mL centrifuge tube. Cell buffer (0.5% BSA + 2 mM EDTA) was added to a final volume of 50 mL, mixed thoroughly, and centrifuged at 400×g for 5 min at room temperature. The supernatant was discarded, and the cell pellet was resuspended in at least 8 volumes of red blood cell (RBC) lysis buffer, followed by incubation on ice for 2 min to lyse residual erythrocytes. After centrifugation at 400×g for 10 min at 4 °C, the supernatant was removed. The PBMC pellet was resuspended in 1 mL of cell buffer, and viable cells were counted using a hemocytometer.

### CD14^+^ monocyte isolation from PBMCs by magnetic bead separation

The PBMC suspension was centrifuged, and the supernatant was discarded. The cell pellet was resuspended at a ratio of 80 μL of cell buffer per 10^7^ cells, followed by the addition of 20 μL of CD14 magnetic beads according to the manufacturer’s instructions. The cell−bead mixture was incubated for 15 min at 4 °C on a thermostatic shaker. Then 500μL cell buffer was added, and the cells were washed by centrifugation. The supernatant was then removed. The cell pellet was resuspended at a ratio of 500 μL of cell buffer per 10⁸ cells. An MS separation column was placed on a magnetic stand and pre−rinsed with 500 μL of cell buffer. The resuspended cell−bead mixture was loaded onto the pre−rinsed column. The column was washed three times with 500 μL of cell buffer per wash. The separation column was then removed from the magnetic stand, and the magnetically retained CD14^+^ monocytes were eluted with 1 mL of cell buffer; the eluate containing the target cells was collected. The collected cell suspension was centrifuged, and the purified monocytes were resuspended in 1 mL of cell buffer. Cell counting was performed using a hemocytometer.

### *In vitro* differentiation of dendritic cells

The purity of CD14⁺ monocytes isolated by magnetic bead separation was verified by flow cytometry. Generation of immature and mature monocyte-derived dendritic cells (moDCs) was performed as previously described (46). Isolated monocytes were resuspended in DC-specific medium and seeded into 6-well plates at a density of 5×10⁵ cells/mL, with 3 mL of cell suspension per well. Recombinant human GM-CSF and IL-4 were added to the culture at final concentrations of 100 ng/mL and 50 ng/mL, respectively. After gentle mixing, plates were incubated in a cell culture incubator following confirmation of cell morphology, density, and distribution under an inverted microscope. On day 3, half of the culture medium was replaced, and GM−CSF and IL−4 were replenished to maintain final concentrations of 100 ng/mL and 50 ng/mL, respectively. Immature dendritic cells (imDCs) were harvested on day 5. For the induction of mature dendritic cells (mDCs), IFN−γ and LPS were added to the culture at a final concentration of 10 ng/mL each, and cells were incubated for an additional 12–24 h.

### Flow cytometry analysis

Cells were collected, centrifuged, and the supernatant discarded. The cell pellet was washed with 1 mL of cell buffer and centrifuged again. After removing the supernatant, cells were resuspended in 100 μL of cell buffer, incubated with 5 μL of Human TruStain FcX at room temperature for 10 min to block Fc receptors. Cells were stained with a panel of optimally diluted cell surface antibodies for flow cytometry analysis, together with 0.1 μL of Fixable Viability Dye APC eFluor 780 per tube to exclude dead cells. The antibodies used were as follows: CD14 (FITC, clone 63D3, 1:100), CD80 (BV510, clone L307.4, 1:100), CD86 (APC, clone IT2.2, 1:100), CD83 (Super Bright 436, clone HB15E, 1:100), CD40 (FITC, clone 5C3, 1:100), CD197 (PE, clone G043H7, 1:100), HLA-DR (Super Bright 600, clone LN3, 1:100), CD206 (PerCP-eFluor 710, clone 19.2, 1:100), and CD45 (Alexa Fluor 700, clone 2D1, 1:100) (**Supplementary Table 3**). The mixture was incubated at 4 °C for 15–30 min under shaking and light-protected conditions. Following the addition of 1 mL cell buffer, cells were centrifuged and washed twice using the same protocol. After removing the supernatant, cells were resuspended in 250 μL Fixation/Permeabilization solution and incubated at 4 °C for 20 min with shaking for fixation and permeabilization. Following fixation and permeabilization, 1 mL of Perm/Wash buffer was added, and the cells were centrifuged and washed twice. The supernatant was aspirated, and the cell pellet was resuspended in 100 μL Perm/Wash buffer. Appropriate intracellular flow cytometry antibodies were then added, and the mixture was incubated at 4 °C for 15–30 min with shaking under light-protected conditions. Thereafter, 1 mL of Perm/Wash buffer was added, and the cells were centrifuged and washed twice. The supernatant was discarded, and the cells were resuspended in 200 μL of cell buffer. Flow cytometry analysis was performed using a Cytek Aurora flow cytometer, and the gating strategy was performed as follows: briefly, cells were gated to exclude autofluorescence, and viable CD45⁺ cells were identified based on CD45 expression and viability staining. MoDCs populations were then defined as CD83⁺CD86⁺ cells, followed by analysis of the expression levels of the indicated markers. All experiments were performed in three independent biological replicates.

### CFSE-based phagocytosis assay

One vial of the CFSE Cell Proliferation Kit was reconstituted by adding 18 μL of dimethyl sulfoxide (DMSO). PBS was preheated to 37 °C and then added to the reconstituted CFSE solution to a final volume of 20 mL, resulting in a working concentration of 5 μmol/L. Target cells were resuspended in the diluted CFSE working solution and incubated at 37 °C for 20 min in the dark. Complete medium was added at a volume five times that of the CFSE solution, and incubation was continued at 37 °C for 5 min to quench residual dye. The cell suspension was centrifuged, the supernatant was discarded, and the cell pellet was resuspended in 200μL of complete medium. Flow cytometry analysis was performed using a BD C6 flow cytometer.

### Monocyte-derived dendritic cells migration assay

Transwell inserts were placed in 24-well plates. The upper and lower chambers were loaded with 200 μL and 800 μL of serum−free RPMI 1640 medium, respectively, followed by equilibration for 15 min. DCs were washed three times with serum−free RPMI 1640 medium and adjusted to a density of 2.5×10⁵ cells/mL. CCL19 was added to the lower chambers at a final concentration of 100 ng/mL, and the prepared moDCs suspension was then added to the upper chambers. The plate was incubated in a cell incubator for 48 h. After incubation, the lower chambers were washed twice with PBS, fixed with 1 mL 4% paraformaldehyde at room temperature for 15 min, and rinsed twice with ddH₂O. Subsequently, the cells were stained with 1 mL hematoxylin for 10 min and washed twice with ddH₂O. Transwell membranes were carefully excised and mounted with glycerol-gelatin. Migrated cells were counted in 5 randomly selected microscopic fields, and the average number was determined.

### Image analysis and statistical analysis

Five high-power fields (400X magnification) were randomly selected from each section. The number of positive cells in each field was counted, and the mean value of the five fields was calculated. Image-Pro Plus software was used to quantify the number of positive cells in tumor tissues, paracancerous tissues, and normal control tissues, respectively, followed by appropriate statistical analyses. The number of positive cells in tumor tissues was compared with that in normal control tissues to evaluate statistical differences. For comparisons of means between two independent samples with normal distribution and homogeneous variances, the independent-sample t-test was used. For comparisons among multiple groups, one-way analysis of variance (ANOVA) was performed. Pearson correlation analysis was used for correlation assessment of normally distributed data. For data that did not conform to normality or homogeneity of variance, the rank-sum test was applied, and Spearman correlation analysis was used for correlation analysis. Survival analysis was performed using the Log-rank test. The significance level was set at p < 0.05. All statistical analyses were performed using GraphPad Prism and SPSS software. Graphs were generated using GraphPad Prism 7 or the R programming language. Statistical significance was defined as follows: *p < 0.05, **p < 0.01, and ***p < 0.001.

## Results

### Distribution profile of IgG4 and dendritic cells in tumor tissues

**Figure 1:**
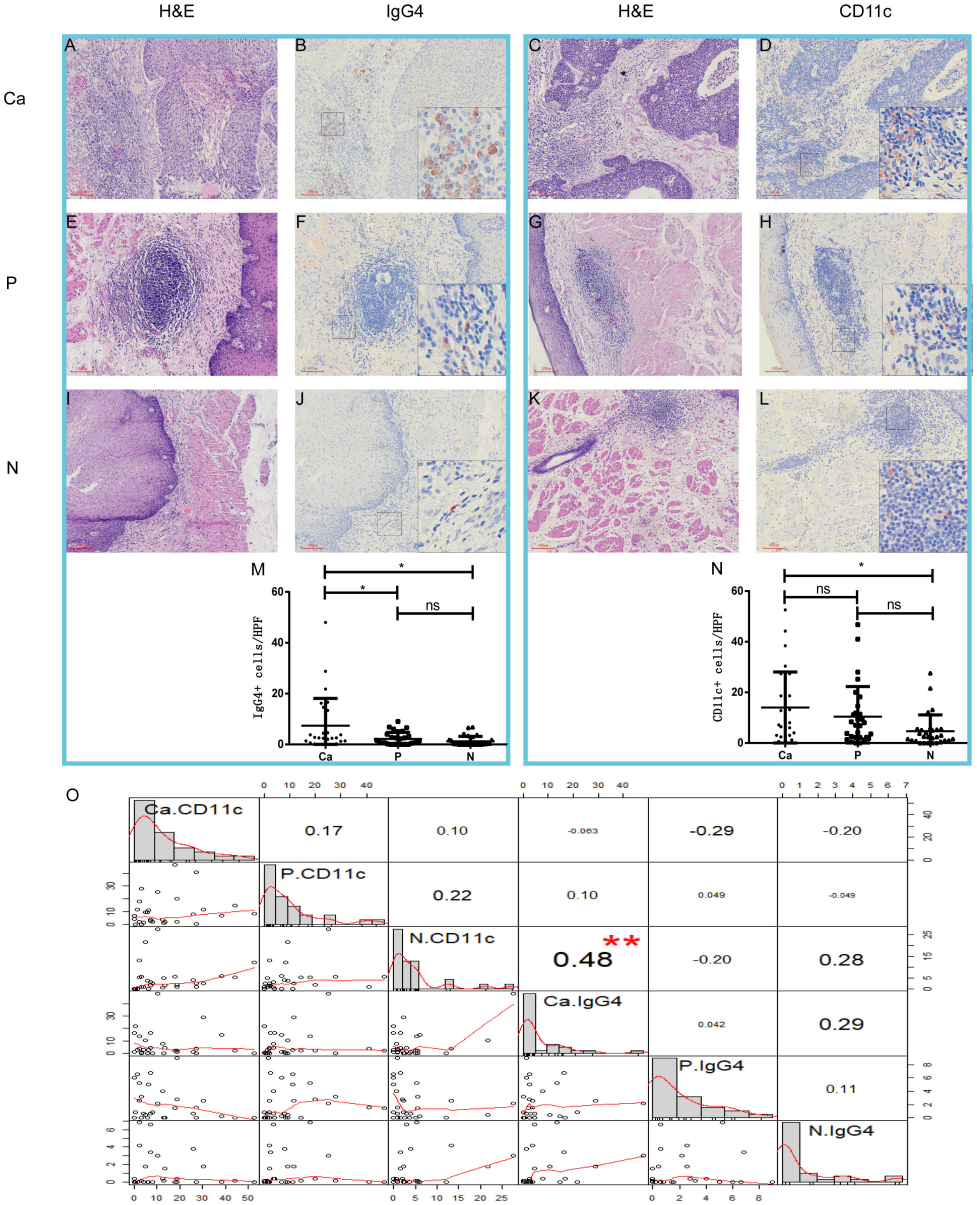
IgG4 and CD11c Expression in Esophageal Cancer and Para-cancerous Tissues **(A–L)**. Tissue sections were arranged from top to bottom as tumor (Ca), para-cancerous (P), and normal control (N) tissues. Left panels: IgG4 staining and corresponding H&E staining; right panels: CD11c staining and corresponding H&E staining. Scale bar =100μm. Insets at the bottom right of IHC images represent 400× magnifications of the boxed regions observed under 10× magnification. **(M, N)** Scatter plots showing the numbers of IgG4- and CD11c-positive cells per high-power field (HPF) in the indicated tissues (n = 29). Data are presented as the mean ± SD. **(O)** Correlation analysis between CD11c and IgG4 expression. Histograms of CD11c or IgG4 expression are shown on the diagonal. The red line represents the fitting curve, and Spearman’s rank correlation coefficient (r) is shown in the upper right corner. *p < 0.05, **p < 0.01, ns, not significant.

In the present study, we evaluated the distribution of IgG4 and DCs in esophageal cancer tissues and paired adjacent non-cancerous tissues. Immunohistochemical staining showed that IgG4 expression was significantly upregulated in tumor tissues compared with para-cancerous and normal control tissues, which was consistent with our previous findings (11). Similarly, CD11c expression was markedly increased in tumor tissues relative to normal tissues, with statistically significant differences (**Figures 1A–N**), suggesting enhanced accumulation of DCs in the tumor microenvironment. To further explore the relationship between IgG4 levels and DC infiltration, we performed correlation analyses based on the counts of IgG4-positive and CD11c-positive cells in tumor, para-cancerous, and normal control tissues, with a correlation matrix constructed to illustrate these associations. As presented in **Figure 1O**, rank correlation analysis revealed a significant positive correlation between CD11c expression in normal control tissues and IgG4 expression in tumor tissues (r = 0.48, p < 0.01).

### *In vitro* co-culture of moDCs stimulated with IgG4

**Figure 2:**
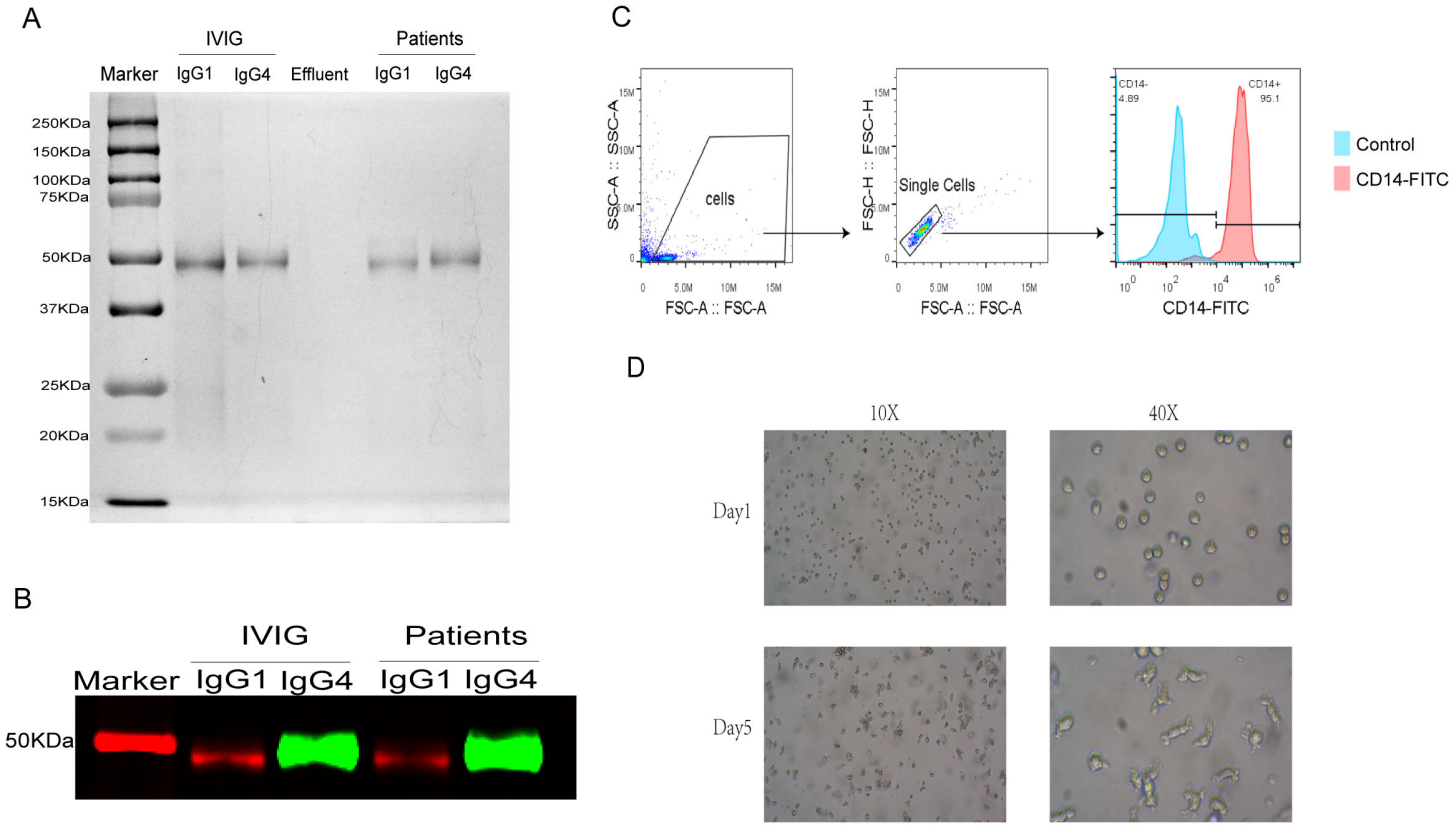
Verification of IgG purification and moDCs generation **(A–D)**. **(A)** Silver staining. Lane sequence: Marker, IgG1, IgG4, flow-through. Purified IgG1 and IgG4 (from both IVIg and esophageal cancer patient serum) showed distinct bands at approximately 50kDa, with no non-specific bands observed. **(B)** Two-color fluorescence Western blotting. Samples included IgG1 and IgG4 from IVIg, as well as IgG1 and IgG4 from serum of esophageal cancer patients. IgG1 was visualized in red, and IgG4 in green. **(C–D)** Isolation of monocytes and differentiation into moDCs. **(C)** Flow cytometric analysis of the purity of magnetically sorted CD14⁺monocytes. **(D)** Morphological characteristics of moDCs. Cells transitioned from a round shape on day 1 to an oval shape with protrusions on day 5.

IgG4 was purified from both therapeutic IVIg derived from healthy donors and serum samples obtained from patients with esophageal cancer. IgG1 and IgG4 subclasses were successfully isolated with high yield and purity. Protein silver staining and Western blot analyses confirmed the high purity of the isolated IgG1 and IgG4 proteins (**Figures 2A, B**). Human peripheral blood monocytes were subsequently isolated using CD14-specific magnetic beads, and the sorting efficiency was validated by flow cytometry (**Figure 2C**). The purified IgG1 and IgG4 subclasses were then used separately to stimulate monocytes *in vitro*. Over the course of cell culture, monocytes exhibited progressive morphological and phenotypic alterations, transitioning from a naïve state to a differentiated and mature phenotype. These observations indicate that IgG1 and IgG4 possess the potential to promote monocyte differentiation (**Figure 2D**).

### Structural characteristics of IgG1 and IgG4 and their solid-phase binding capacity to monocytes

**Figure 3:**
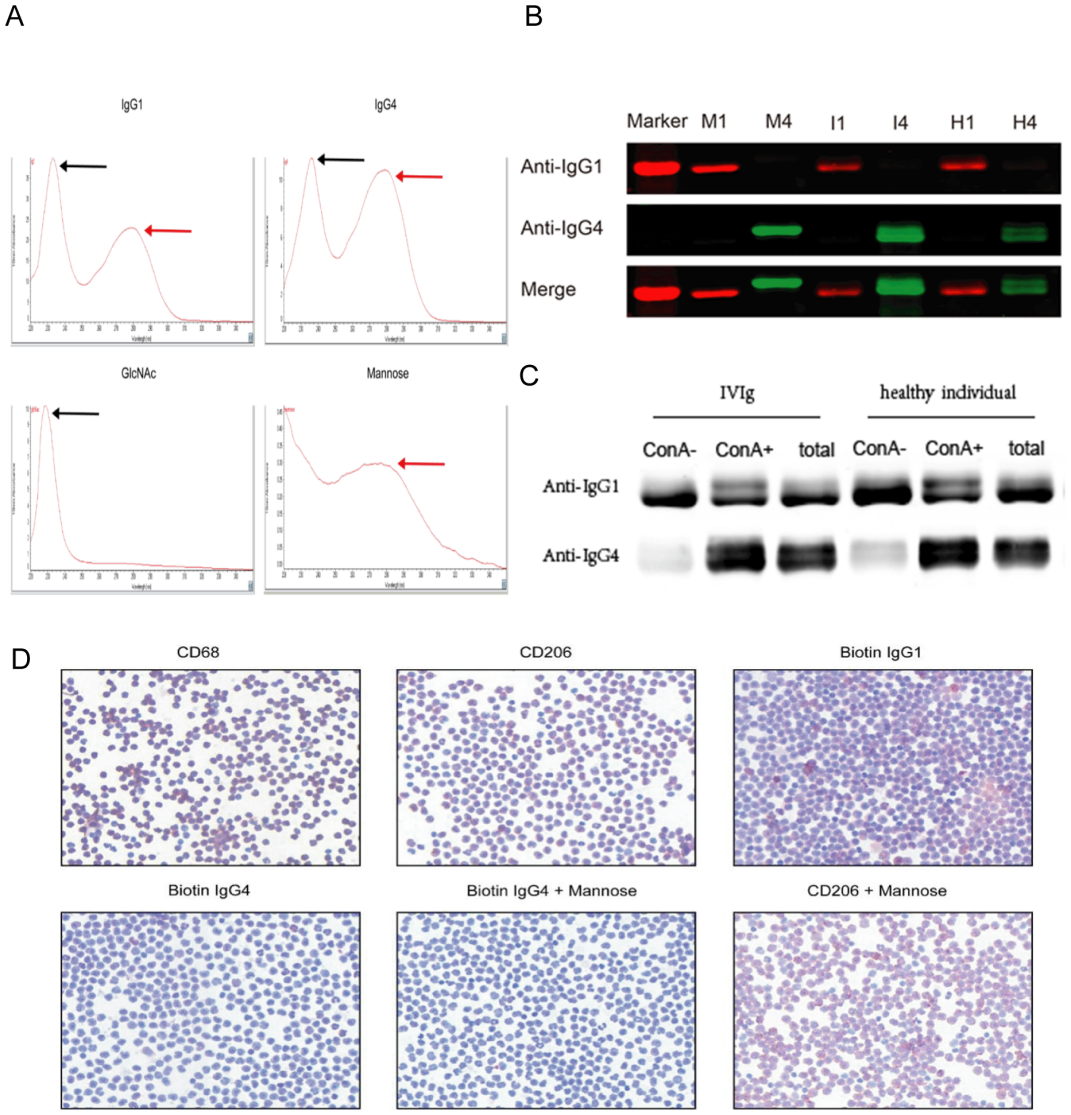
**(A)** Waveform diagrams derived from spectral detection of IgG1 and IgG4 purified from IVIg, as well as N-acetylglucosamine and mannoside. **(B)** M, I, and H denote IgG1 or IgG4 proteins from commercial human myeloma (M), IVIg (I), and healthy human serum (H), respectively. Anti-IgG1 antibody was labeled with red fluorescence, and IgG4 was labeled with green fluorescence. **(C)** Serum IgG from IVIg and healthy volunteers was subjected to lectin affinity chromatography to separate ConA⁺ IgG and ConA⁻ IgG fractions. Western blot staining with anti-IgG1 and anti-IgG4 antibodies revealed that IgG4 was predominantly enriched in the ConA⁺IgG fraction. **(D)** Staining patterns of CD68, CD206, Biotin−IgG1, Biotin−IgG4, Biotin−IgG4 + mannose, and CD206 + mannose in purified healthy human CD14^+^monocytes after slide coating. Positive signals were visualized as reddish-brown using AEC chromogenic staining.

In addition to differences in their amino acid sequences, distinct structural modifications were observed between IgG1 and IgG4. Spectroscopic analysis revealed that the characteristic peaks of IgG1 overlapped with those of N-acetylglucosamine, whereas the spectral profile of IgG4 showed a marked overlap with mannose, suggesting an enrichment of mannose-related structures on IgG4 (**Figure 3A**). In line with these observations, IgG4 displayed a strong binding affinity for concanavalin A (ConA), a lectin highly specific for mannose-containing glycans. Using ConA affinity chromatography, IgG4-enriched proteins were successfully isolated, further verifying the presence of mannose-associated glycosylation on IgG4 (**Figure 3C**). To evaluate the purity and structural characteristics of the isolated immunoglobulins, IgG1 and IgG4 purified from both IVIg and patient serum were compared with commercial IgG4 standards. Fluorescence analysis showed minimal signal overlap between the IgG1 and IgG4 preparations, verifying their high purity and suitability for subsequent functional experiments. Notably, human serum-derived IgG4 exhibited two distinct bands in the heavy−chain region, one of which displayed an approximate 1–2 kDa increase in molecular weight. This observation indicates the presence of unique post−translational modifications on native IgG4, which are absent in IgG1 or commercial myeloma cell−derived IgG4 (**Figure 3B**). Next, to examine their binding capacity to monocytes, purified IgG1 and IgG4 were biotinylated and incubated with freshly isolated PBMC−derived monocytes immobilized on coverslips. Immunocytochemical staining demonstrated robust binding of IgG1 to monocytes, whereas IgG4 showed relatively weak binding activity (**Figure 3D**). These observations suggest that IgG4–monocyte interactions may rely on mechanisms distinct from classical Fc receptor–mediated binding. Furthermore, immunocytochemical staining confirmed high expression of mannose receptors on the surface of monocytes.

### Phenotypic alterations in mo-DCs induced by IVIg and serum-derived IgG1 and IgG4 from patients with esophageal cancer

**Figure 4:**
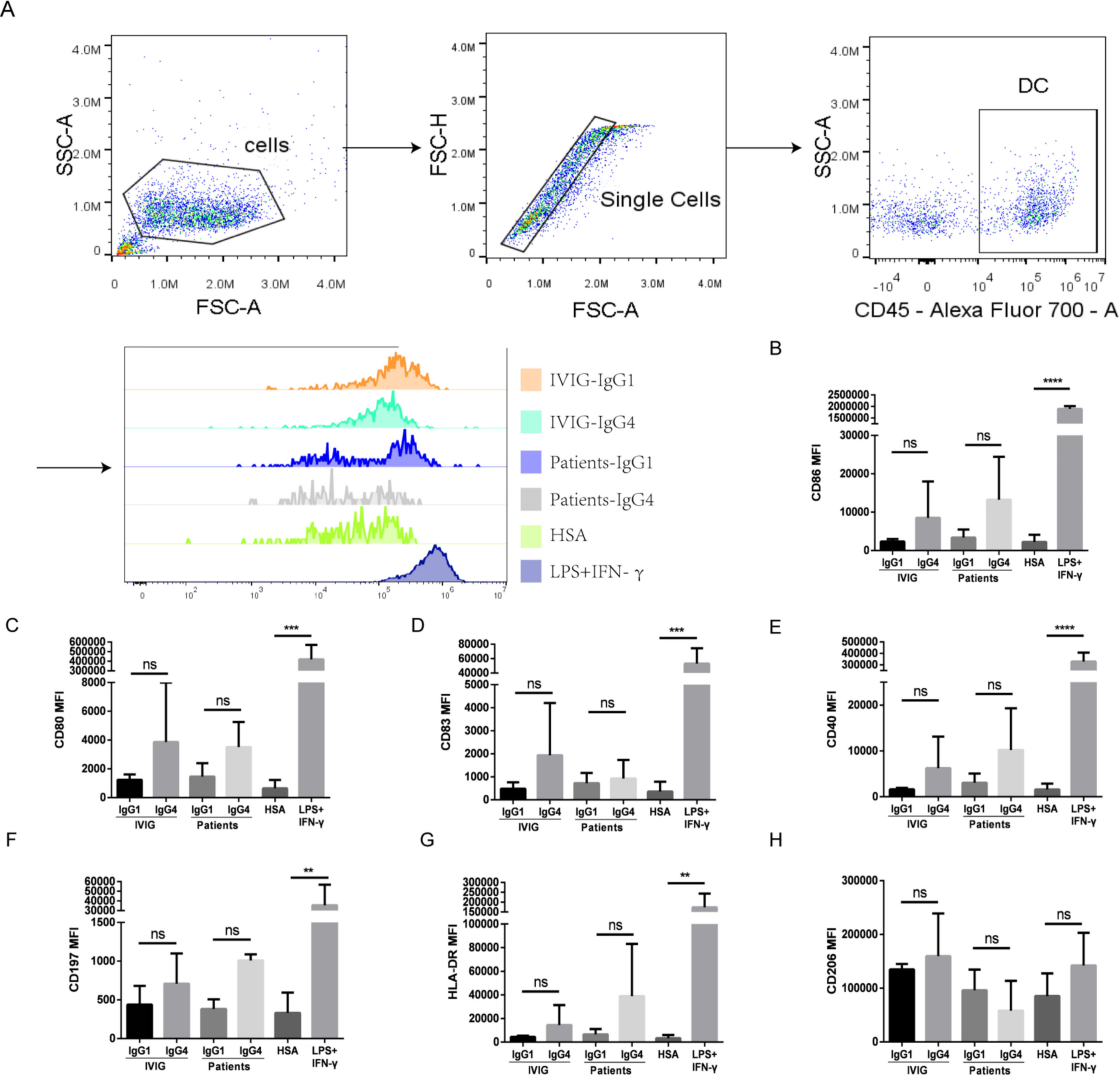
Impacts of IgG1 and IgG4 on moDCs Phenotypic Markers. **(A)** Flow cytometry gating and fluorescence histograms. MoDCs were gated by FSC-A-SSC-A, followed by single-cell gating via FSC-A-FSC-H, and finally identified as CD45-Alexa Fluor 700-positive cells. Middle panels: fluorescence intensity histograms of each marker; right panels: sample groups corresponding to each fluorescence color. **(B–H)** Bar charts showing the geo-MFI of markers (CD80, CD86, HLA-DR, CD40, CD83, CD206, CD197) across groups. Groups: IgG1/IgG4 from IVIg; IgG1/IgG4 from esophageal cancer patient serum; HSA (negative control); LPS + IFN-γ (positive control) (n = 3). **p < 0.01, ***p < 0.001; ns, not significant.

We compared the effects of IgG subclasses derived from esophageal cancer patient serum and healthy donor–derived IVIg on moDCs differentiation and phenotypic changes. Following stimulation of monocyte-to-DC differentiation, moDCs phenotypic markers were analyzed by flow cytometry. Based on cell size, granularity, and viability gating strategies, the viable moDCs population was identified (**Figure 4A**). Flow cytometric analysis revealed that the expression levels of CD80, CD86, CD83, CD40, CD197 (CCR7), and HLA-DR were consistently higher in the IgG4-stimulated group than in the IgG1-stimulated group, although the difference was not statistically significant. This pattern was observed in both the healthy donor–derived IVIg group and the patient serum–derived IgG group, indicating that IgG4 from different sources exerts comparable effects on promoting dendritic cell phenotypic changes (**Figures 4B–H**). Notably, the expression pattern of CD206 differed between the two groups. In the healthy donor–derived IVIg group, CD206 expression was higher in IgG4-stimulated moDCs than in IgG1-stimulated moDCs, although no statistical significance was observed for the p-value. In contrast, in the patient serum–derived IgG group, CD206 expression was higher in the IgG1 group than in the IgG4 group (**Figures 4B–H**), indicating a potential source-dependent divergence in CD206 regulation.

### Impact of IgG4 on phagocytosis and migration of moDCs

**Figure 5:**
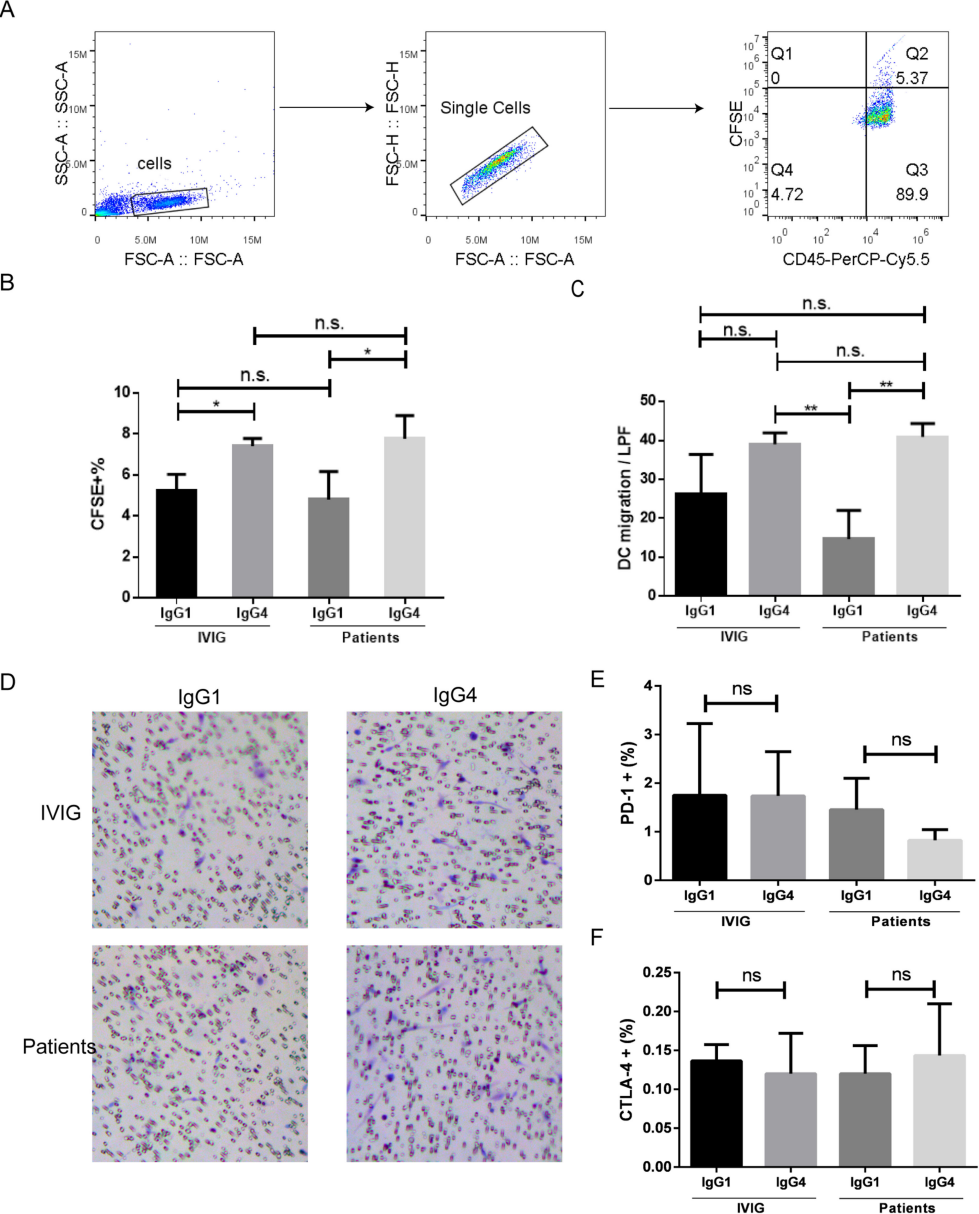
MoDCs Phagocytosis and Migration Experiments **(A)** Flow cytometry gating plots. **(B)** Quantification of phagocytosis rate (Q2 proportion). X-axis: IgG1/IgG4 from IVIg and esophageal cancer patient serum; Y-axis: CFSE-positive rate (n = 4). **(C)** Migrated cell counts of each group (IVIg-derived IgG1/IgG4, patient serum-derived IgG1/IgG4) in 5 low-power fields (n=3). **(D)** Hematoxylin-stained transwell membrane images after moDCs migration; moDCs were identified as hematoxylin-positive cells with protrusions. **(E, F)** X-axis: IgG1/IgG4 from IVIg and patient serum; Y-axis: positive rates of PD-1 and CTLA-4 on T cells, respectively. p < 0.05, p < 0.01, *p < 0.001; ns., no statistical significance (n = 3).

To evaluate the effects of IgG4 on dendritic cell phagocytic capacity, CFSE-labeled ESCC cell lines subjected to repeated heat treatment and liquid nitrogen freeze–thaw cycles were co-cultured with moDCs and analyzed by flow cytometry. After gating viable cell populations and excluding doublets, moDCs that had phagocytosed tumor cells were identified as CD45⁺CFSE⁺ cells in the Q2 quadrant (**Figure 5A**). Quantitative analysis demonstrated that the phagocytic rates of moDCs were significantly higher in both IVIg-derived and patient serum–derived IgG4-stimulated groups compared with their respective IgG1-stimulated controls (**Figure 5B**). Notably, no significant differences were observed between IVIg-derived and patient serum–derived groups within the same IgG subclass, indicating comparable effects of IgG4 from different sources on moDCs phagocytic activity. The migratory capacity of moDCs was assessed using a Transwell assay with CCL19 as the chemotactic stimulus. MoDCs were seeded in the upper chambers, and migrated cells in the lower chambers were quantified in five randomly selected fields (**Figure 5D**). Statistical analysis revealed that patient serum–derived IgG4 significantly enhanced moDCs migration compared with patient serum–derived IgG1. Similarly, IVIg-derived IgG4 induced a higher number of migrated moDCs than patient serum–derived IgG1 (**Figure 5C**). To further investigate whether IgG4-modulated moDCs influenced T cell exhaustion–associated markers, magnetically isolated CD8⁺ T cells were co-cultured with moDCs, and the expression of PD-1 and CTLA-4 was assessed by flow cytometry. No statistically significant differences in PD-1 or CTLA-4 expression were detected among moDCs stimulated with IVIg-derived or patient serum–derived IgG1 and IgG4 (**Figures 5E, F**).

## Discussion

Our data showed that CD11c expression was elevated in tumor tissues compared with normal control tissues, and IgG4 expression in tumor tissues was higher than that in paracancerous and normal control tissues. Previous reports have indicated that CD11c⁺ DCs exhibit enhanced capacity for tumor infiltration and antigen presentation (47, 48), which led us to focus on this specific DC subset. Similar high expression patterns of CD11c⁺ DCs have been documented in breast cancer, metastatic melanoma, head and neck squamous cell carcinoma (49, 50). We further analyzed the expression levels of IgG4 and CD11c, and explored their statistical correlation. As indicated in **Figures 3**, **4**, the correlation between IgG4 infiltration and CD11c⁺ DC levels in tumor tissues lacked statistical significance, possibly due to numerous outliers, suboptimal tissue morphology of these outliers on H&E staining, and insufficient sample size. On the other hand, IgG4 infiltration in tumor tissues was correlated with CD11c⁺ DC expression in normal control tissues, which hints at a potential regulatory effect of IgG4 on moDCs migration. A notable limitation of this study is the relatively small cohort of tissue specimens. That said, our previous analysis of over 100 paraffin samples yielded a consistent trend: IgG4 expression in tumor tissues was higher than that in adjacent normal tissues. Moreover, our prior work has verified that high IgG4 infiltration in ESCC and correlates with unfavorable prognosis.

This study showed that IgG4 and DC expression were abnormally increased in tumor-associated tissues, and IgG4 in tumor tissues was positively correlated with DCs in normal control tissues. Although IgG4-positive and CD11c-positive cells were both increased in ESCC tissues compared with adjacent non-tumor tissues, the current data do not establish a direct functional relationship between IgG4 and dendritic-cell infiltration within the tumor microenvironment. The association observed in adjacent non-tumor tissue may reflect local immune or inflammatory features; however, in the absence of a consistent within-tumor association and additional mechanistic validation, this observation should be considered exploratory. Also, the number of tissue samples in this study was limited, and the correlation analysis showed relatively weak statistical support; therefore, these findings require further validation in larger patient cohorts.

For *in vitro* cell experiments, primary DCs isolated directly from tissues are limited by their low abundance and technical challenges in extraction. Therefore, most studies generate DCs via induced differentiation of alternative cell sources, including umbilical cord blood cells, peripheral blood CD34⁺ hematopoietic stem cells, and peripheral blood monocytes (51). Peripheral blood monocytes are the most preferred source because of their easy acquisition and high yield, and the induced cells are defined as moDCs. Despite the phenotypic and functional discrepancies between moDCs and primary DCs (28, 52), moDCs are still competent in mediating immune regulation in the tumor microenvironment. Moreover, moDCs express CD11c, which renders them a suitable cell model for this study. The morphological changes shown in **Figure 2D** confirmed the successful differentiation of moDCs. In this study, IgG4 was sourced from two origins: IVIg and tumor patient serum, with IgG1 (a canonical effector molecule) used as the control. Following IgG purification, the protein was incubated with pretreated ESCC cells to form tumor immune complexes, and the impacts of IgG1 and IgG4 on moDCs phenotype and function were evaluated.

In the DC phenotype assay (**Figure 4**), HSA, as the negative control, and LPS plus IFN-γ, as the positive control, provided reliable baseline references. This design is consistent with the study by Matthias Wölfl et al. (53), which compared DC phenotypic responses under different stimulatory conditions. Among the analyzed DC markers, CD80, CD86, and HLA-DR are commonly used to assess antigen presentation- and co-stimulation-related phenotypic features; CD206 reflects antigen recognition and pattern sensing; CD40 and CD83 are indicative of DC maturation; and CD197 is associated with DC migration. The results showed that the positive control group had significantly higher marker expression than all other groups, while no statistical differences were detected among the IgG groups (IVIg-derived vs. patient-derived, IgG1 vs. IgG4). Although the mean geo-MFI of IgG4 groups was higher than that of IgG1 groups, the small sample size and high intra-group variation may have masked the actual significant difference, highlighting the need for larger cohorts in future studies. As reported by Yaron Carmi et al. (45), co-stimulation with additional factors can enhance IgG-mediated DC activation (54), and CD11c⁺ DCs are the primary subset mediating anti-tumor immunity in mouse tumor models.

In DC functional assays, as shown in **Figures 5A, B**, the phagocytic rate of CFSE-labeled fixed tumor cells was higher in IgG4 groups than in IgG1 groups, regardless of IgG source, whereas no difference was observed between IVIg-derived and patient-derived subgroups of the same IgG subclass. This suggests that the effects of IgG4 depend on its intrinsic structural and functional features rather than its source, and tumor-derived IgG4 may exert similar functions. The migration assay confirmed that IgG4 promoted moDCs migration, which aligned with our hypothesis; again, no difference was found between IVIg-derived and patient-derived IgG subsets. The cells with protrusions also verified the successful differentiation of monocytes into DCs. We also preliminarily explored the antigen-presenting function of moDCs. To investigate immunosuppressive effects, we detected PD-1 and CTLA-4 expression on T cells via flow cytometry, but no significant differences were found among groups. Additionally, ELISA results showed no difference in IFN-γ levels, implying that the tested IgG subsets may not affect T cell function directly. Although migration and phagocytosis are important indicators for evaluating certain functional properties of DCs, they only reflect partial functional features of moDCs and cannot substitute for direct evidence of antigen presentation, T-cell priming, cytotoxic T-cell activation, or *in vivo* anti-tumor immune effects. Therefore, the potential regulatory role of IgG4 in DC function and anti-tumor immunity requires further validation through more systematic functional assays, including antigen-presentation assays, T-cell priming assays, cytokine release analyses, tumor antigen-specific T-cell response assays, and animal model studies. The present *in vitro* moDC experiments were based on a limited number of independent biological replicates and selected phenotypic and functional readouts. Although these data suggest differences between IgG1- and IgG4-treated moDCs, they are insufficient to fully define dendritic-cell maturation status, subset identity, antigen-presenting competence, or immunoregulatory function. Future studies should include larger numbers of independent donors, more comprehensive flow-cytometric gating strategies, immature and mature DC markers, DC subset-specific markers, cytokine profiling, receptor-blocking experiments, antigen-presentation assays, and T-cell functional readouts to determine whether IgG4-associated changes in moDCs have biological relevance *in vivo*.

At the cellular level, our data confirmed that IgG4 complexes loaded with ESCC promoted moDCs phagocytosis and migration, while no differences in T cell suppression after antigen presentation were observed, which contradicts previous literature reports (55). Hiltbold et al. (56) reported that tumor glycoproteins CEA and MUC1 are trapped in DC endosomes after endocytosis, inhibiting further antigen processing and uptake of other antigens, thus preventing efficient antigen presentation to T cells. Furthermore, non-activated (immature) DCs can present antigens to T cells and induce immune tolerance via T cell deletion or differentiation of regulatory/suppressive T cells (57–60). This study primarily focused on changes in maturation-associated phenotypic markers of moDCs after IgG1/IgG4 treatment, rather than directly demonstrating alterations in their antigen-presenting capacity or immunoregulatory function. We acknowledge that changes in selected surface markers alone, particularly in the context of a relatively small sample size, are insufficient to fully define DC maturation status, subset identity, or functional competence. More rigorous DC characterization and functional validation will require a more comprehensive flow-cytometric gating strategy, combined assessment of immature and mature-state markers, DC subset-specific markers, and functional assays such as antigen uptake, T-cell stimulation, and cytokine secretion analyses, which should be further strengthened in future studies.

Future studies should further optimize experimental strategies to more comprehensively elucidate the immunoregulatory role of IgG4. Several approaches may strengthen the mechanistic resolution of IgG subclass–mediated moDCs regulation. First, the inclusion of additional co-stimulatory signals, such as Poly(I:C), TNF-α, or CD40L, may amplify functional differences between IgG1 and IgG4 and better reflect the complexity of inflammatory cues present in the tumor microenvironment. Second, flow cytometric profiling of migration-related receptors on moDCs derived from tumor, paracancerous, and normal tissues, together with immunohistochemical analysis of chemokine expression within the tumor microenvironment, would provide further insight into the mechanisms underlying IgG4-associated moDCs migration and spatial distribution. In parallel, more refined classification of moDCs subsets across different tissue compartments may help delineate subset-specific responses to IgG4. Additionally, it will be important to verify whether the observed effects of IgG4 on moDCs phenotype and function are consistent between *in vitro* tumor immune complex models and the native tumor microenvironment *in vivo*. Comprehensive assessment of T cell activation markers and tumor cell–killing capacity following moDCs-mediated antigen presentation will further clarify whether IgG4-modulated moDCs shape downstream adaptive immune responses. Finally, the establishment of appropriate animal models that recapitulate the tumor immune microenvironment will be essential to validate the role of IgG4-mediated moDCs regulation in tumor immunity and to determine its functional relevance *in vivo*.

The unique glycosylation pattern of IgG4 identified in this study may influence its biological functions. Structurally, IgG4 displays high similarity to asymmetrically glycosylated IgG and may be involved in the regulation of placental immune tolerance (61). The biological functions of immunoglobulins are not solely determined by their amino acid sequences, but are also critically regulated by post-translational glycosylation (62). Compared with IgG1, IgG4 exhibits distinct glycosylation patterns and structural heterogeneity, which may be associated with its unique functional properties. Previous reports have suggested that IgG4 is enriched in noncanonical glycan modifications, including high-mannose–associated structures, potentially conferring immunomodulatory functions that differ from those of classical effector IgG subclasses (63). Importantly, mannose-containing glycans can be recognized by C-type lectin receptors, among which the mannose receptor CD206 is highly expressed on monocytes and dendritic cells and plays a key role in antigen uptake, cell migration, and immune regulation (64, 65). Thus, the unique glycosylation features of IgG4, particularly mannose-related modifications, may enable IgG4 to influence dendritic cell function through non–Fc receptor–mediated pathways, such as CD206-dependent interactions. This glycan-associated mechanism provides a plausible molecular basis for our observation that IgG4 enhances dendritic cell phagocytosis and migration without directly promoting T cell effector responses. This study has several limitations. Comprehensive glycan profiling, site-specific glycosylation analysis, enzymatic deglycosylation assays, receptor-blocking experiments, and gain- or loss-of-function validation were not performed. Therefore, the precise mechanisms by which specific IgG4 glycoforms, particularly mannose-related or high-mannose structures, may contribute to the regulation of dendritic cell function remain to be further clarified through systematic glycomic analyses and functional validation studies. In our preliminary deglycosylation experiments, PNGase F was used to remove N-linked glycans under denaturing conditions, and the deglycosylation efficiency was confirmed by Western blotting. However, owing to the exploratory nature of the current study and technical limitations in generating biologically functional deglycosylated IgG preparations suitable for cell-based assays, these mechanistic experiments were not further extended in the present work, thereby limiting our ability to directly assess the biological effects of deglycosylated IgG on dendritic cells. Future studies will focus on optimizing enzymatic or chemical glycan-modification strategies under native or cell-compatible conditions, including the use of mannosidase-related approaches, to clarify the specific role of mannose-rich glycan structures in regulating dendritic cell maturation, antigen presentation-related marker expression, and immune-regulatory functions. Collectively, these findings indicate that IgG4 glycosylation features may be associated with its immunological effects in the tumor immune microenvironment, although the underlying molecular mechanisms and functional significance remain to be further clarified.

Although IgG4 has been widely regarded as an immunosuppressive or immunomodulatory antibody subclass in the tumor microenvironment (9, 13, 66), our *in vitro* experiments revealed that IgG4 induced a higher degree of phenotypic changes, phagocytic capacity, and migratory activity in moDCs compared with IgG1. This seemingly paradoxical observation highlights an important conceptual distinction between IgG4 mediated dendritic cell phenotypic changes and IgG4 mediated tumor immune evasion. Previous studies have demonstrated that enhanced dendritic cell phagocytosis or migration does not necessarily translate into productive antigen presentation or T cell priming, particularly in the absence of sufficient inflammatory cues or co-stimulatory signals (24, 67, 68). In such contexts, dendritic cells may retain tolerogenic or regulatory properties despite exhibiting features commonly associated with activation. Moreover, specific dendritic cell subsets play a critical role in the generation of cell-associated antigen–specific cytotoxic T lymphocyte responses (69). Therefore, the IgG4-mediated potential enhancement of moDCs functional parameters observed in this study is more likely to reflect qualitative modulation of dendritic cell functional states rather than direct promotion of anti-tumor immune effector responses. The molecular mechanisms underlying this regulatory effect of IgG4 warrant further investigation.

## Conclusion

In conclusion, both IgG4 and CD11c are upregulated in esophageal squamous cell carcinoma, and their expression patterns exhibit a positive association. Distinct from the canonical effector immunoglobulin IgG1, IgG4 enhances the phagocytic and migratory capacities of dendritic cells, and these effects are independent of IgG origin, whether tumor-derived or non–tumor-derived. These findings indicate that IgG4 plays a potential immunomodulatory role in shaping moDCs function within the tumor immune microenvironment.

## Data Availability

The original contributions presented in the study are included in the article/**Supplementary Material**. Further inquiries can be directed to the corresponding authors.
