# Supplementary material for: Exploratory characterization of IgG1/IgG4 glycosylation and monocyte-derived dendritic cell responses in esophageal squamous cell carcinoma
Source: Front Immunol. 2026 Jun 18;17:1832782. doi: 10.3389/fimmu.2026.1832782 (PMC13323231; doi:10.3389/fimmu.2026.1832782)
Supplement: Supplementary file 1 [file Table1.docx]

Supplementary Table 1.

| **IHC Antibody** | **Species origin** |  | **Manufacturer** |
| --- | --- | --- | --- |
| anti-human CD11c | Rabbit | - | Abcam |
| anti-human CD11c | Mouse | - | Santa Cruz |
| anti-human IgG1 | Mouse | - | Abcam |
| anti-human IgG4 | Rabbit | - | ZSGB-BIO |
| anti-human IgG4 | Rabbit | - | Abcam |

Supplementary Table 2.

| **WB secondary Antibody** | **Species origin** | **Conjugated fluorophore** | **Manufacturer** |
| --- | --- | --- | --- |
| anti-mouse IgG 680 | Goat | IRDye 680RD | LI-COR |
| anti-rabbit IgG 800 | Goat | IRDye 800CW | LI-COR |

Supplementary Table 3.

| **Flow Cytometry**  **Antibody** | **Species origin** | **Conjugated fluorophore** | **Manufacturer** |
| --- | --- | --- | --- |
| anti-human CD14 | Mouse | FITC | BioLengd |
| anti-human CD197 | Mouse | PE | BioLengd |
| anti-human CD206 | Mouse | PerCP-eFluor 710 | Thermo Fisher |
| anti-human CD40 | Mouse | FITC | Thermo Fisher |
| anti-human CD40 | Mouse | PE | BioLengd |
| anti-human CD45 | Mouse | Alexa Fluor 700 | Thermo Fisher |
| Anti-human CD45 | Mouse | PerCP-Cy5.5 | 4A BIOTECH |
| anti-human CD45RA | Mouse | FITC | BioLengd |
| anti-human CD45RO | Mouse | PE | Thermo Fisher |
| anti-human CD62L | Mouse | FITC | BioLengd |
| anti-human CD8 | Mouse | FITC | BioLengd |
| anti-human CD8 | Mouse | APC | BD Biosciences |
| anti-human CD80 | Mouse | Brilliant Violet 510 | BD Biosciences |
| anti-human CD83 | Mouse | Super Bright 436 | Thermo Fisher |
| anti-human CD86 | Mouse | Brilliant Violet 711 | BD Biosciences |
| anti-human HLA-DR | Mouse | Super Bright 600 | Thermo Fisher |
| anti-human HLA-DR | Mouse | APC-H7 | BD Biosciences |
| anti-human IgG | Rabbit | FITC | Boster |
| anti-human PD-1 | Mouse | PE | BioLengd |
| anti-human CTLA-4 | Mouse | APC | BioLengd |
